# Case report: Uterine leiomyoma with fumarate hydratase deficiency

**DOI:** 10.3389/fmed.2024.1391978

**Published:** 2024-05-09

**Authors:** Diana Bužinskienė, Dominyka Grinciūtė, Mindaugas Šilkūnas, Evelina Šidlovska

**Affiliations:** ^1^Faculty of Medicine, Vilnius University, Vilnius, Lithuania; ^2^Clinic of Obstetrics and Gynecology, Institute of Clinical Medicine, Faculty of Medicine Vilnius University, Vilnius, Lithuania; ^3^National Center of Pathology, Affiliate of Vilnius University Hospital Santaros Klinikos, Vilnius, Lithuania

**Keywords:** case report, hereditary leiomyomatosis and renal cell carcinoma, skin leiomyomatosis, uterine leiomyomatosis, fumarate hydratase

## Abstract

Hereditary leiomyomatosis and renal cell cancer syndrome is a rare autosomal dominant disease caused by mutations in the fumarate hydratase gene. The syndrome is characterized by skin leiomyomatosis, uterine leiomyomatosis, and renal cell carcinoma. Herein, we report a case of fumarate hydratase deficient leiomyoma. The patient was a young female presenting with large uterine leiomyoma and multiple kidney angiomyolipomas. The report presents the chosen treatment and the challenges of differential diagnosis.

## Introduction

Hereditary leiomyomatosis and renal cell cancer (HLRCC) syndrome is a rare autosomal dominant disease caused by mutations in the fumarate hydratase (FH) gene. Skin leiomyomatosis, uterine leiomyomatosis, and type II renal cell carcinoma are the characteristic triad of symptoms for the disease (1). The average age of the patients is 30 years old. Syndromic uterine leiomyomas are usually multiple and large, while the main complaints of the patients are heavy, irregular, and painful menstruation. Even though, both skin and uterine leiomyomas are benign, type II renal cell carcinoma is an aggressive type of cancer, thus, it is essential to diagnose FH-deficient leiomyomas histologically and to investigate the patient further (2). In this case report we present a 29-year-old patient with FH-deficient leiomyoma.

### Narrative

In 2022, a 29-year-old patient was referred to a gynecologist for a routine checkup regarding a uterine leiomyoma. The uterine fibroid was first identified 8 years ago after a computer tomography (CT) scan of the pelvis and abdomen due to angiomyolipoma of the right kidney. At the time, the tumor measured 3 cm. According to the gynecological anamnesis, menstrual cycles were regular (cycle duration 27 days, bleeding lasting 6 days), and occasionally painful. No history of sexual intercourse. The patient has not experienced previous gynecological illnesses or surgeries. In 2014, an abdominal CT scan was performed which showed multiple kidney angiomyolipomas along with a gigantic angiomyolipoma (AML) of the right kidney. The same year right kidney resection was performed, along with arterial embolization of the AML. We could not acquire the histological images as kidney resection was performed in another hospital. However, we did receive a pathologist’s conclusion: “Microscopic examination revealed a tumor composed of fat tissue, smooth muscle cells, and thick blood vessels with hyaline walls. Consistent with the diagnosis of kidney angiomyolipoma.”

**Figure 1:**
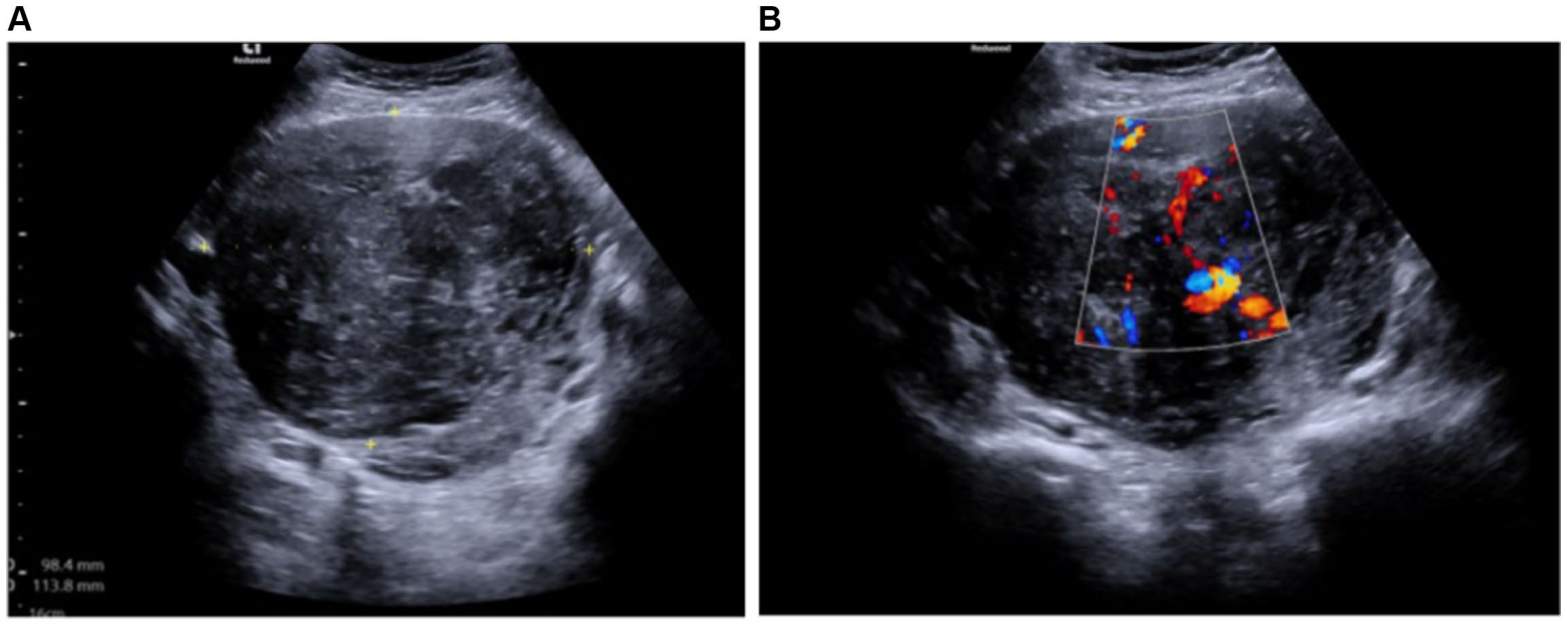
**(A)** Mixed echogenicity uterine tumor measuring 9.84 × 11.38 cm (transabdominal ultrasound). **(B)** Tumor with an active blood flow (transabdominal ultrasound).

During the gynecological examination, the abdomen was non-tender, and a large mass was palpable in the pelvic region. Transabdominal and transrectal sonoscopy was performed, revealing a large-sized mass in the pelvic area measuring 9.84 × 11.38 cm, displaying mixed echogenicity with active blood flow (likely uterine fibroid or sarcoma) (Figure 1). Ovaries were not visualized due to the size of the mass. No free fluid or additional formations were observed.

**Figure 2:**
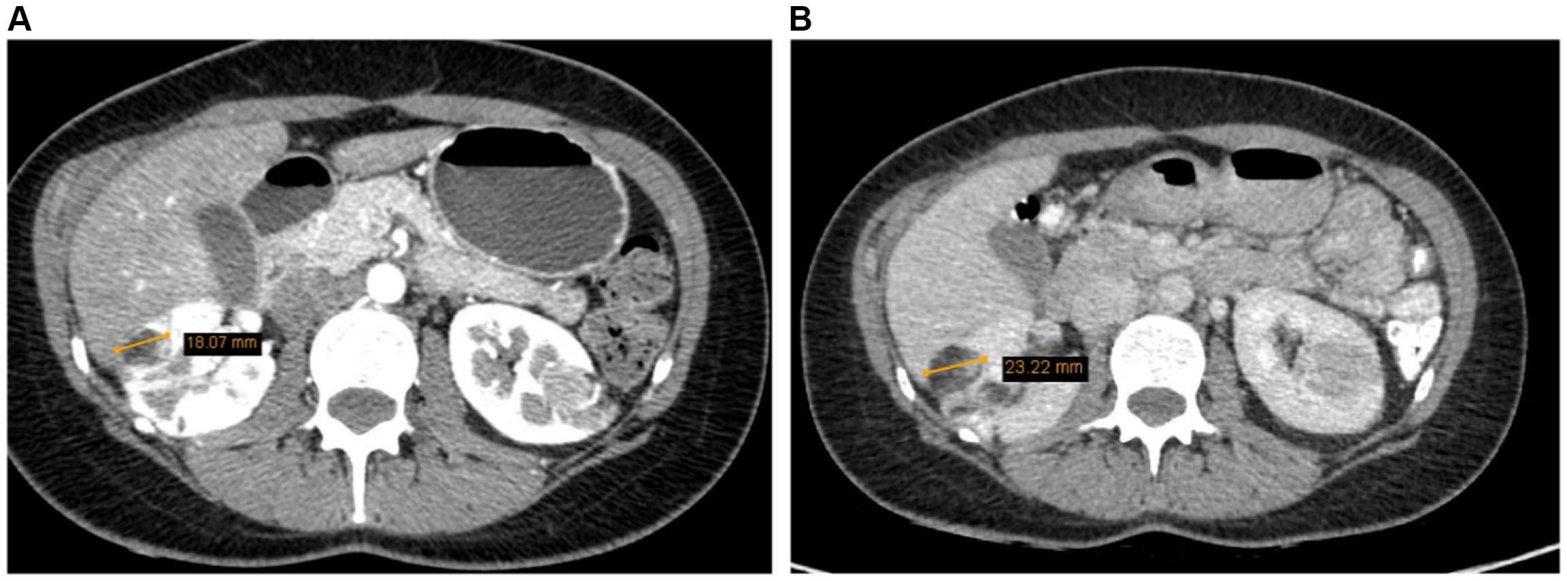
**(A)** Right kidney AML 1.8 cm (2017). **(B)** Right kidney AML 2.3 cm (2022).

A whole-body (CT) was recommended for further clarification of the diagnosis and to assess the potential spread of the neoplastic process. CT scans of the neck, chest, abdomen, and pelvic organs were performed using intravenous and oral contrast. The images were compared with the abdominal CT conducted in 2017, evaluating the post-kidney resection view. The kidneys appeared to be in their normal position, and the collecting system was not dilated. The upper third of the right kidney was resected, showing multiple hypodense cortical defects (likely scar changes). Multiple angiomyolipomas were observed in the parenchyma of both kidneys. In terms of dynamics (compared to 2017), there was an increase in the size of AML – the largest angiolipoma on the front surface of the right kidney has grown from 1.8 cm to 2.3 cm. No hemorrhages were observed in the angiomyolipomas. The contrast was appropriately excreted by both kidneys, freely flowing into the bladder (Figure 2).

**Figure 3:**
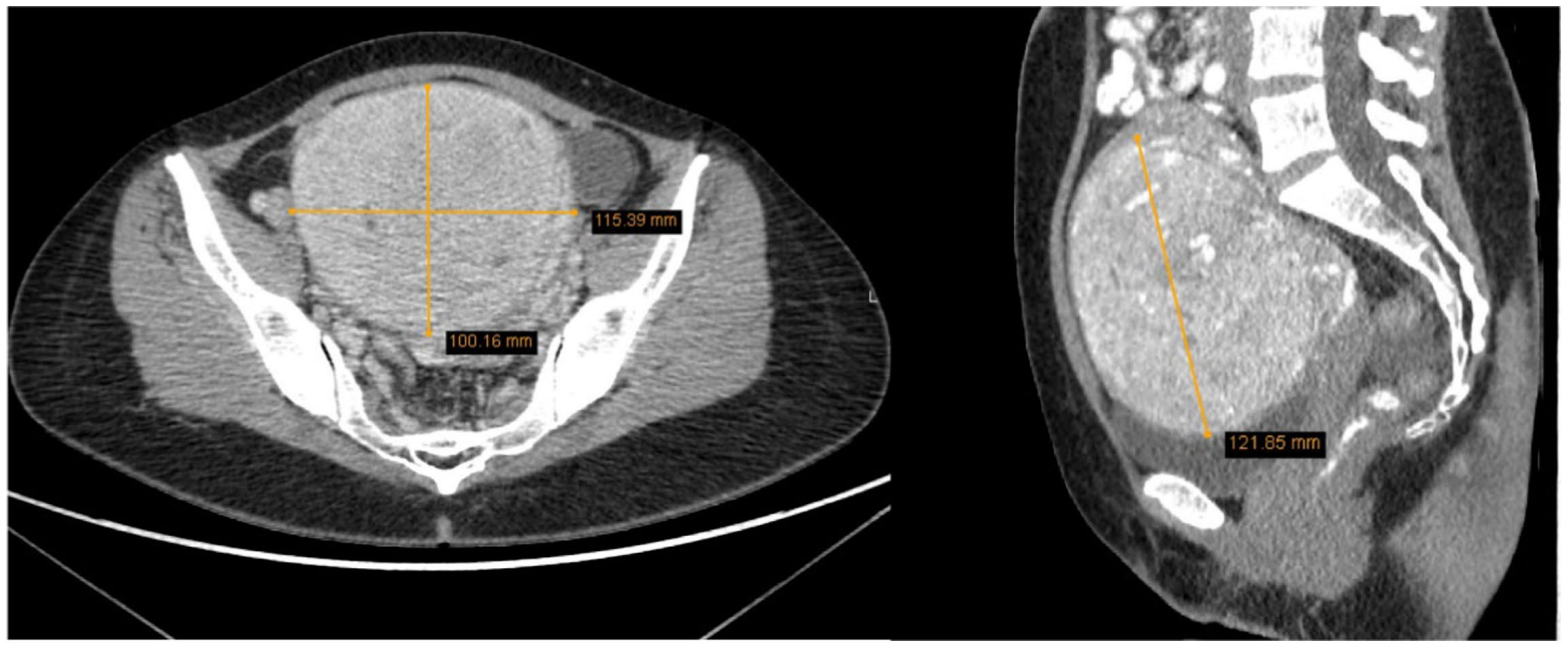
The tumor measured 10.0 × 11.5 × 12.2 cm, displacing surrounding structures but has not metastasized (2022 CT scan).

The uterus was anteverted and displaced to the left due to the mass. The tumor had areas of lower density and accumulated contrast. It measured 10.0 × 11.5 × 12.2 cm and closely interacted with surrounding structures, but had not metastasized. Enlarged ovarian veins were visible on both sides, the ovaries were also displaced due to the size of the tumor. In the right ovary, corpus luteum was visible along with several functional cysts up to 1.7 cm (Figure 3). There was no free fluid in the pelvic cavity. According to the radiologist’s conclusion, there were no clear signs of malignancy in the uterine tumor on the CT scan, and it was most likely a large uterine leiomyoma. The recommended treatment for the patient was surgery – laparotomic myomectomy.

Due to the large dimensions of the uterine tumor, a laparotomic myomectomy was performed for the patient. During the operation, the fibroid was enucleated, its integrity intact. The patient lost 1700 mL of blood during the surgery, leading to blood transfusion and fresh-frozen plasma transfusion. Uterine contraction-inducing drugs were administered, including 10 units of Oxytocin intravenously and 10 units in a drip infusion. After evaluating clinical symptoms, objective examination, and test results, as well as surgical findings, a clinical diagnosis of intramural leiomyoma of the uterus was established.

**Figure 4:**
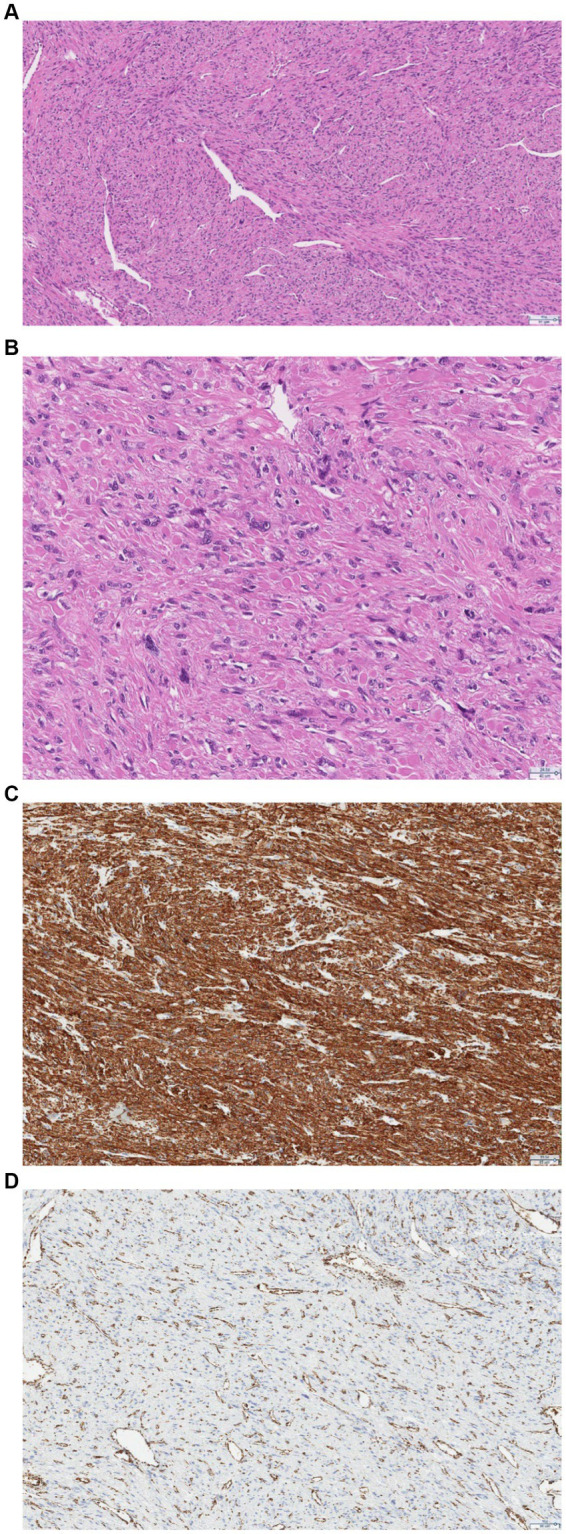
**(A)** Pathologic examination confirmed features of FH deficiency: hemangiopericytoma-like or “staghorn” vasculature. **(B)** At higher magnification, the nuclei are round-to-oval with prominent eosinophilic nucleoli, perinuclear clearing, and eosinophilic cytoplasmic globules. **(C)** Immunohistochemistry shows positive staining for smooth muscle actin. **(D)** Immunohistochemistry shows a loss of FH staining with retained expression in endothelial cells.

After the operation, the removed uterine tumor was sent for histological examination. Microscopic examination revealed a tumor composed of spindle cells with round to elongated nuclei with macronucleoli with perinucleolar halos. Cytoplasmic eosinophilic globules and staghorn vessels were observed. Some scattered multinucleated, giant cells with bizarre nuclei were also present. Immunohistochemically, there was a loss of fumarate hydratase. Therefore, the final pathological diagnosis was leiomyoma with fumarate hydratase loss (Figure 4).

Leiomyoma with fumarate hydratase loss may be associated with hereditary leiomyomatosis and renal cell carcinoma syndrome. In the presence of this syndrome, there is an increased risk of developing cutaneous and uterine leiomyomatosis, as well as renal cell carcinoma (Type II papillary carcinoma). Therefore, the patient should undergo regular outpatient care with a gynecologist (every 6–12 months) and consult with an oncogenetic specialist. The patient underwent oncogenetic testing using next-generation sequencing, which did not identify pathological mutations in the FH, TSC1, and TSC2 genes. There may be a mutation in intronic noncoding regions (about 10%), however, it is not detected by standard genetic testing methods.

As the patient was diagnosed with a giant uterine leiomyoma with FH loss and growing renal angiomyolipomas, it has been decided to further discuss the patient’s examination and treatment strategies in a multidisciplinary consilium. The multidisciplinary team involved an urologist, a radiologist, a chemotherapy oncologist, and a radiotherapist. It was decided that, currently, there were no indications for surgical treatment of renal angiomyolipomas, and it was recommended to continue monitoring the patient every 6 months performing radiologic examination of kidneys.

### Patient perspective

The patient, following the surgical treatment she underwent, is feeling well, without any gynecological complaints. Currently, her menstrual cycle is regular and she feels no abdominal or pelvic pain. The surgical incision is healing well, and no pathological changes were observed in the uterus and both adnexa. The patient has expressed she is satisfied with the recommended treatment. In April 2024, a multidisciplinary consilium convened once again to review the patient’s case. Given the absence of discernible changes, it was determined that the patient should continue to be monitored without the necessity for kidney surgery. Furthermore, since no pathological mutation of the FH gene was detected, the prognosis remains favorable. The patient reported no alterations in their quality of life during the evaluation.

## Discussion

Considering the presented case, it is important to discuss the role of FH gene. Fumarate hydratase is an enzyme encoded by FH gene located in chromosome 1. This gene encodes both mitochondrial and cytosolic isoforms of the enzyme. The enzyme is responsible for the conversion of fumarate to L-malate during the tricarboxylic acid cycle (3). There can be homozygous or heterozygous germline gene mutations. In both cases the conversion of fumarate to L-malate is disrupted, leading to the accumulation of fumarate in the cytosol and mitochondria (4). Homozygous germline mutations are very rare and lead to an autosomal recessive disorder – fumaric aciduria. Patients with these mutations rarely survive into adolescence. Heterozygous germline mutations, causing loss of FH, predispose to the inheritance of autosomal dominantly inherited leiomyomatosis and renal cell carcinoma syndrome. In this case, cellular metabolism is disrupted, and it is one of the causes of cancer (3). In the case of heterozygous germline mutations, the function of FH is lost, and an excess of fumarate begins to accumulate in cells, initiating an oncogenic cascade. Due to the accumulation of fumarate in the body, compensatory changes are observed, such as the inhibition of oxidative phosphorylation in mitochondria, leading to a shift towards anaerobic glycolysis. Glucose-6-phosphate metabolism through the pentose phosphate pathway, resulting in a decrease in the overall adenosine triphosphate (ATP) levels. These changes are characteristic of the Warburg effect, described as a process where glucose consumption increases, and lactate production occurs, as cells generate energy anaerobically independent of a sufficient oxygen supply. Such metabolism is one of the distinguishing features of cancer cells (4, 5). The excess of fumarate also stabilizes the hypoxia-inducible factor, which under normal conditions is constantly hydroxylated by prolyl hydroxylase to prevent its surplus and avoid promoting hypoxia. Stabilization of this factor results in oxygen deficiency, initiating the transcription of genes responsible for angiogenesis and cell growth. FH deficiency is also associated with an increase in free radicals, which additionally inhibits prolyl hydroxylase and deepens hypoxia. As cytosolic FH is responsible for DNA damage repair it is also impaired in the absence of FH (3).

It is also important to discuss the recommended treatment for leiomyomas and examination of the patients when HLRCC syndrome is suspected. Leiomyomas may be treated conservatively or surgically. In the presented case, the tumor is large and symptomatic, thus requiring surgery. Since the patient is of reproductive age and uterus preservation is important, myomectomy was chosen over hysterectomy (6). Even though, monitoring was not an option in this situation due to the large size of the leiomyoma and fertility preservation was important, there may be future complications with the patient’s pregnancy after the surgery. The main concerns after myomectomy are prolonged first labor stage and elevated rate of induction of labor. Myomectomy may also be associated with an elevated rate of emergency cesarean section due to uterine rupture. However, in La Verde et al. (7) prospective study no uterine ruptures were observed comparing women’s pregnancies after myomectomy and with no previous history of this surgery. As for the choice of open surgery, in this particular case, the consideration of ultra-minimally invasive surgery was deemed unfeasible despite its recognized merits, including superior aesthetic outcomes and pain tolerance achievable through a reduced trocar count (8). This decision stemmed primarily from the considerable size of the leiomyoma, which presented a complex surgical challenge even for an experienced surgical team. Hysteroscopic myomectomy was ruled out as it is better suited for submucosal fibroids, rather than intramural as was in the presented case (9). Laparoscopic surgery decreases postoperative pain, offers faster recovery, minimal scar formation, and improves quality of life. Nonetheless, laparotomic surgery allows easier manipulation in the surgical area which was important due to the size of the tumor (10).

Uterine leiomyomatoma with FH deficiency may be independent of HLRCC syndrome, however, it is required to further investigate the patient with this diagnosis. As required the patient had an appointment with an oncogenetic specialist and no pathological mutations were observed (2). The patient has also been followed closely by an urologist due to multiple kidney AMLs and no signs of malignancy were ever noted. However, there is a possibility that not all relevant mutations have been identified yet. In the case of HLRCC syndrome, the patient should be monitored by a dermatologist, a gynecologist, and a urologist. Skin leiomyoma is usually benign, however, sometimes tumors can cause pain and discomfort. In such cases, surgical excision, cryoablation, or laser treatment may be considered. There is no consensus on how often patients should be monitored by a dermatologist. Syndromic uterine leiomyomas usually tend to be large and symptomatic, thus, often requiring surgery before the age of 30. Yearly gynecologist follow-ups are recommended (2). Renal cell carcinoma is the most dangerous of the triad due to its aggressiveness and metastasis risk. It is recommended to perform abdominal CT or magnetic resonance tomography (MRT) with gadolinium-based contrast each year starting from 10/11 years of age, as there are reported cases of patients developing renal cell cancer at the age of 10 (2, 11). The initial method should be MRT due to its higher precision and absence of radiation exposure. It is recommended to perform the scanning with slices of 1–3 mm thickness (12).

Another limitation of this case report – the patient was never evaluated by a dermatologist, as another common symptom of HLRCC syndrome is skin leiomyomatosis (2, 12, 13). This limitation also leads to a possible differential diagnosis error. HLRCC along with tuberous sclerosis complex are both hereditary kidney cancer syndromes. Multiple kidney angiomyolipomas along with skin hamartomas or angiofibromas, and neurological conditions, such as seizures, autism spectrum disorders, or cognitive disability, are characteristic to tuberous sclerosis complex (14, 15). In this case, multiple kidney AMLs and their size are concerning. The average age for non-syndromic AMLs is 40 years and more, they usually measure 1–4 cm and grow 0.19 cm on average per year. However, AMLs associated with tuberous sclerosis have an earlier onset (20–30 years), averagely measuring 3.5–19,3 cm and growing about 1.25 cm a year (16). Therefore, the patients’ AMLs do remind of AMLs associated with tuberous sclerosis. However, the data is insufficient to confirm tuberous sclerosis diagnosis as the patient has not been evaluated by either a dermatologist or a neurologist. Both consultations with a dermatologist and a neurologist were advised; nonetheless, the patient declined to pursue them on the grounds of perceiving an absence of neurological or dermatological complaints.

Another reason why the presence of AMLs is concerning – they may be masking kidney cancer. Angiomyolipoma is a benign tumor composed of fatty and muscular tissue, as well as blood vessels. It is the most common among benign kidney tumors, diagnosed in approximately 30 out of 100,000 patients, more frequently in women (17, 18). Clinically, its course does not differ from renal cell carcinoma – AML is usually asymptomatic. However, if symptoms occur, they usually manifest as a triad: lower back pain, hematuria, and palpable masses in the kidney projection. Contrast-enhanced abdominal CT or MRT is usually sufficient to diagnose the tumors as definitive diagnosis can only be acquired through the tumor biopsy which is rarely performed due to the high risk of bleeding. Radiologically AMLs are classified into three categories: abundant fatty tissue, minimal fatty tissue, and AML with no detectable fatty tissue. The distinguishing feature of angiomyolipoma is fatty tissue, which can be observed in CT or MRT, making AML with abundant fatty tissue easily recognizable and safe to monitor by repeating imaging studies every 6–12 months. The challenge arises when AML with minimal or no fatty tissue is found; in such cases, CT and MRT are insufficient for differentiation between AML and renal cell carcinoma. It is recommended to biopsy such angiomyolipomas to avoid missing kidney cancer. Additionally, if a kidney mass with fatty intersperses and calcifications is observed, the diagnosis of renal cell carcinoma is much more likely, as calcifications are not characteristic of AML (17). Thus, in this patient’s case monitoring every 6 months is essential along with biopsy if any minimal or no fatty tissue tumors are noted.

## Conclusion

This case report confirms the importance of a thorough investigation of the patient. According to the clinical and surgical findings and correctly chosen diagnostic algorithm the most likely diagnosis is FH-deficient leiomyoma unassociated with HLRCC syndrome. However, a slim chance of two other potential diagnoses remains. It may be HLRCC syndrome and the pathological mutations have not been yet identified. The other possibility may be that the patient has FH-deficient leiomyoma unassociated with HLRCC along with tuberous sclerosis complex. Nonetheless, with the available medical history, clinical and surgical findings healthcare specialists have chosen the best treatment option.

## Data availability statement

The original contributions presented in the study are included in the article/supplementary material, further inquiries can be directed to the corresponding authors.

## Ethics statement

Written informed consent was obtained from the individual(s) for the publication of any potentially identifiable images or data included in this article.

## Author contributions

DB: Conceptualization, Methodology, Project administration, Supervision, Writing – review & editing. DG: Investigation, Methodology, Writing – original draft. MŠ: Supervision, Validation, Writing – review & editing. EŠ: Resources, Supervision, Writing – review & editing.
